# Reference intervals for the echocardiographic measurements of the right heart in children and adolescents: a systematic review

**DOI:** 10.1186/1476-7120-12-3

**Published:** 2014-01-29

**Authors:** Carolina E Lemmer (Hunsinger), Mark E Engel, John C Stanfliet, Bongani M Mayosi

**Affiliations:** 1 Department of Medicine, University of Cape Town and Groote Schuur Hospital, Observatory, Cape Town, South Africa; 2 National Health Laboratory Service and Department of Chemical Pathology, University of KwaZulu-Natal, Durban, South Africa

**Keywords:** Echocardiography, Reference ranges, Normal values, Right ventricle, Right atrium, Tricuspid, Pulmonary, Vena cava, Paediatric

## Abstract

**Background:**

Transthoracic echocardiography is the primary imaging modality for the diagnosis of right ventricular (RV) involvement in congenital and acquired heart diseases. There is increasing recognition of the contribution of RV dysfunction in heart diseases affecting children and adolescents, but there is insufficient information on reference intervals for the echocardiographic measurements of the right heart in children and adolescents that represent all the continental populations of the world.

**Objective:**

The aim of this systematic review was to collate, from published studies, normative data for echocardiographic evaluation of the right heart in children and adolescents, and to identify gaps in knowledge in this field especially with respect to sub-Saharan Africans.

**Methods:**

We performed a systematic literature search to identify studies of reference intervals for right heart measurements as determined by transthoracic echocardiography in healthy children and adolescents of school-going age. Articles were retrieved from electronic databases with a combination of search terms from the earliest date available until May 2013.

**Results:**

Reference data were available for a broad range of variables. Fifty one studies out of 3096 publications were included. The sample sizes of the reference populations ranged from 13 to 2036 with ages varying from 5 to 21 years. We identified areas lacking sufficient reference data. These included reference data for determining right atrial size, tricuspid valve area, RV dimensions and areas, the RV % fractional area change, pulmonary artery pressure gradients and the right-sided haemodynamics, including the inferior vena cava dimensions and collapsibility. There were no data for sub-Saharan African children and adolescents.

**Conclusion:**

Reliable reference data are lacking for important echocardiographic measurements of the RV in children and adolescents, especially for sub-Saharan Africans.

## Introduction

Transthoracic echocardiography is the primary imaging modality for the diagnosis of right ventricular (RV) failure [1-4]. Besides aiding in the diagnosis of conditions such as arrhythmogenic right ventricular cardiomyopathy, pulmonary embolism, and RV infarction, echocardiography plays a critical role in the diagnosis of congenital heart diseases where the RV often serves as the main pumping chamber [5-8].

The absence of reference intervals for cardiac structures in children and adolescents is an important problem [6]. Publications dedicated to the echocardiographic study of the right heart, especially on reference values of the structure and function of the right heart in children and adolescents are scarce. Many of the previous studies had limited sample sizes, and were conducted predominantly in North American and European populations. Thus there is potential for interpretation errors when assessing African children, given that environmental, social, economic and other factors may influence the anthropometric standards of a population [9].

Systematic reviews provide rigorous, objective evidence to assess the literature through the use of a pre-specified protocol and access a variety of database search engines. The use of explicit, systematic methods in reviews limits bias and reduces the effects of chance, providing more reliable results [10]. The aim of this systematic review was to collate, from published studies, normative data for echocardiographic evaluation of the right heart in children and adolescents, in order to identify gaps in knowledge in this field, especially with respect to the available of information on sub-Saharan African children.

## Methods

### Types of studies

This review considered all publications reporting reference values for the right heart in healthy children and adolescents determined by echocardiography.

### Types of reference individuals

Participants included school children and adolescents with no history or echocardiographic evidence of heart disease. No exclusion was set on sample sizes of the studies.

### Inclusion and exclusion criteria

Inclusion criteria:

•Age range from 5 to 21 years.

•Documentation of age and/or an indicator body size for reference individual(s). Indicators of body size included height and/or weight and/or body mass index (BMI) and/or body surface area (BSA).

•Presentation of results as one reference value and/or -interval for the whole sample or, in relation to age and/or body size.

•Availability of English version of paper in the case of foreign-language articles.

Exclusion criteria:

•Preterm infants.

•Deceased participants (autopsy studies).

•Participants with known cardiovascular disease.

•Measurements taken at high altitude ≥2400 metres above sea level.

•Age groups including adults (without subgroups for participants younger than 22 years).

•Presentation of reference values that had already been included in a previously published article.

### Search strategy and selection of studies

**Figure 1:**
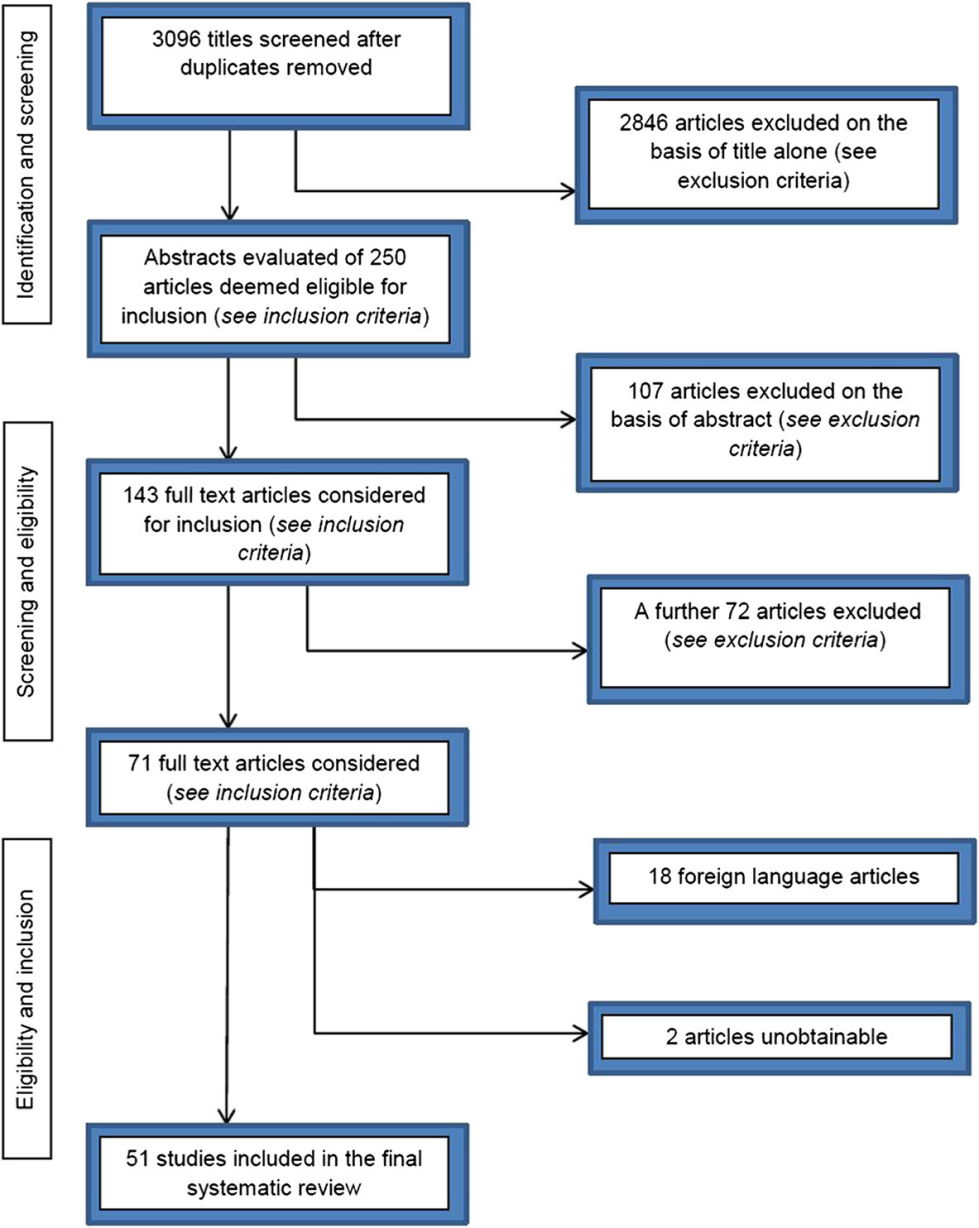
Flow diagram of the article selection process.

Figure 1 details the process by which articles were selected for inclusion.

The scope of search aimed to include all published work dating back to the start of routine echocardiography. We searched the Pubmed and ISI Web of Knowledge databases with a combination of the following search terms: ECHOCARDIOGRA* [Title/Abstract] AND ("NORMAL VALUES” [Title/Abstract]) OR ("NORMAL RANGES" [Title/Abstract]) OR ("REFERENCE VALUES"[Title/Abstract]) OR ("REFERENCE RANGES" [Title/Abstract]) OR ("REFERENCE INTERVALS" [Title/Abstract]) from the earliest date available until May 2013. Limits included *humans*. Categories included *Imaging and Radiology*. This process was complemented by reviewing citations, searching with Google Scholar, expert referrals and hand-searching. Additional articles were included as they became available.

We combined the outputs from the databases *PubMed* and *ISI Web of Knowledge* using a referencing program, Endnote® (Version X5; Thomson Reuters). After duplicate entries were removed, titles of citations were screened for possible inclusion. The titles of potentially relevant studies were reviewed, after which the abstracts and full text articles were examined for possible inclusion. We attempted to find an English copy of the full text article for all of the selected abstracts. Articles were graded as eligible, potentially eligible, or not eligible based on the inclusion and exclusion criteria.

### Data extraction and analysis

We extracted the following data: the year of study, sample size, age range, study setting, sample selection methods, measurements and methods of measurements onto a data extraction form. For each age-group, the sample size with reported summary statistics (i.e., mean, median, centiles, standard deviation, confidence intervals, or standard error) for measurements were also documented.

### Quality assurance

The systematic review was conducted according to the methods of the Cochrane Collaboration [11]. We included all available published reference values of echocardiographic evaluation of the right heart in children and young adults in an attempt to minimise publication bias.

## Results

From the 3096 publications retrieved from the databases, we identified 51 studies for inclusion in this report. 2 846 articles were excluded on the basis of title alone, while a further 107 abstracts and 72 articles failed to meet the inclusion criteria. 18 of the 19 foreign language articles were excluded because no English translation was available. We were able to include data from the remaining foreign language article as the sub-headings of the tables containing reference data, were in English. Two articles were unobtainable.

### Description of studies

The majority of included studies were conducted in clinical settings (such as hospitals, clinics, or medical centres), and research laboratories. 17 studies did not specify the setting. 15 of the included 51 studies were published before the year 2000, while 36 were published from 2000 onwards. The oldest publication dates back to 1977 [12]. Twenty two studies were from reference populations in North or South America, 17 from European countries, 8 from Asia, 1 from the Middle East, 1 from Australia and 1 from north Africa (Egypt).

The sample size of the reference populations ranged from 13 [13] to 2 036 [14]. The ages of the reference populations ranged from 5 to 21 years.

In 24 studies, the reference population consisted of individuals that were referred for echocardiography to exclude cardiac disease [6,8,9,15-35]. Nine of the studies presented the reference data of volunteers [12,14,22,36-41]. There were two studies sampling reference participants from wards [38,42], two studies sampled participants from a database(s) [43,44] and one study reported reference values of normal control group [13]. Forteen studies were unclear as to the method of sample selection [45-58]. None of the included studies had a population-based design.

Table 1 summarizes the sample characteristics of all 51 included studies [6,8,9,12-33,35-60]. As the right heart examination should include a measure of the right atrial (RA) and RV size and structure, RV systolic function and pulmonary artery (PA) pressures, [1] our findings are presented in Table 2 (published reference data for right heart size/volume), Table 3 (published reference data for right heart morphology), Tables 4, 5, and 6 (published reference data for RV- and valve function),and Table 7 (published reference data for right heart haemodynamics).

**Table 1:** Sample characteristics of the 51 included studies

**Author**	**Year**	**Sampling methods**	**Sample size**	**Age-range**	**Age-categories**	**Sample size per age-category**
Ayabakan [15]	2003	REF, V, O	72	3 d - 16 y	4	18
Boettler [45]	2005	U	129	1 d - 16.9 y	1	129
Bonatto [9]	2006	REF	595	1 m - 144 m	*(Data presented according to BSA)*	
Cantinotti [59]	2013	*A review containing nomograms*				
Cui [16]	2008	REF	593	1 d - 18 y	1	593
Daubeney [17]	1999	REF, U	125	1 m - 207 m	*(Regression equations and Nomograms)*	
Eidem [36]	1998	V	152	3 y - 18 y	1	152
Eidem [18]	2004	REF	325	1 d - 18 y	5	55 - 81
Frommelt [19]	2002	REF	141	3 d - 18 y	2	27; 114
Goebel [37]	2006	V	45	5 y – 23 y	1	45
Gutgesell [20]	1991	U	70	1 d – 18 y	*(Data presented according to BSA)*	
Hanseus [46]	1988	U	120	3 d – 15.5 y	*(Regression equations and Nomograms)*	
Harada [21]	2000	REF	48	7 d - 18 y	1	48
Hershenson [60]	2010	U	16	5.8 ± 1.7 y	1	16
Hui [44]	2010	O	103	3 - 18 yrs	4	19 - 29
Ichida [38]	1987	O, V	173	0 d – 15 y	7	8 - 21
Innelli [22]	2009	REF, V	40	10 y - 19 y	1	40
Ishii [8]	2000	REF	150	30 d - 18 y	1	150
Jin [23]	1997	REF	108	7 d - 17 y	5	12 - 29
Kampmann [14]	2000	REF, V	2036	1 d - 18 y	*(Data presented according to BSA / weight)*	
Kapusta [47]	2000	V	160	4 y - 17.9 y	1	160
King [24]	1985	REF	103	1 d – 15 y	*(Data presented according to BSA)*	
Koestenberger [26]	2009	REF	640	1 d - 18 y	22	18 - 47
Koestenberger [25]	2012	REF	860	1 m - 18 y	29	8 - 83
					*(Graphically according to age and BSA)*	
Kutty [27]	2013	REF	153	<1 - 20 y	*5*	*21 - 24*
					*(Contour plots as a function of age)*	
Lange [48]	1983	U	185	Birth - 15 y	*(Data presented according to BSA / weight)*	
Lester [39]	1987	V	202	25 d - 23 y	*(Data presented according to BSA)*	
Matsui [49]	2007	U	22	1.6 - 10.8 y	1	22
McQuillan [43]	2001	O	856	< 20 y	1	856
Moiduddin [13]	2010	O	13	5.7 y ± 1.8 y	1	13
Mori [50]	2004	U	396	Birth - 19 y	3	130 - 135
Norgard [51]	1992	U	15	6 y - 16 y	1	15
Nunez-Gil [28]	2011	REF	405	0 d - 18 yrs	9	15 - 77
					*(Graphically according to weight, height and BS*	
Pettersen [29]	2008	REF	782	1 day - 18 y	*(Data presented according to BSA)*	
Rafeiyian [30]	2005	REF	100	1 m - 15 y	4	9 - 46
Roberson [31]	2007	REF	634	1 d - 18 y	*(Data presented according to BSA)*	
Roberson [53]	2007	U	308	1 d - 18 y	6	41 - 77
Sarnani [32]	2009	U	179	0.02 m - 19 y	1	179
Seguela [33]	2012	REF	50	2 m - 18 y	1	50
Shedeed [40]	2010	V	60	5 - 15 y	1	60
Singh [41]	1994	V	78	2 m - 50 y	4	7 - 47
Stines [54]	2011	U	16	5.7 ± 1.7 y	1	16
Suleymanoglu [6]	2007	REF	213	15 d - 15 y	*(According to weight and graphically for age)*	
Van der Hulst [55]	2011	U	123	1 m - 18 y	5	*(Not presented in tables)*
Vignola [12]	1977	V	17	3 y - 17 y	1	17
Weidemann [56]	2002	U	33	4 - 16 y	1	33
Wessel [57]	1985	U	30	*(Children – ages not stated)*	*(Data presented according to weight)*	
Yusuoka [34]	1999	REF	99 30(TDI)	7 d - 22 y	*(Data presented graphically according to age)*	
Zhendong [58]	1998	U	88	3 y - 12 y	2	41; 47
Zilberman [35]	2005	REF	748	Birth - 18 y	*(Data presented according to BSA)*	

REF, referred to exclude cardiac disease; V, volunteers; O, other; d, day(s); y, year(s); U, unknown; m, month(s); BSA, body surface area; BS, body surface; TDI, tissue Doppler imaging.

**Table 2:** Published reference data for right heart size/volume

**Measurement**	**Boettler **[45]	**Bonatto **[9]	**Daubeney **[17]	**Gutgesell **[20]	**Hanseus **[46]	**Ichida **[38]	**Innelli **[22]	**Jin **[23]	**Kampmann **[14]	**King **[24]	**Lange **[48]	**Lester **[39]	**Matsui **[49]	**Norgard **[51]	**Pettersen **[29]	**Shedeed **[40]	**Singh **[41]	**Suleymanoglu **[6]	**Vignola **[12]	**Wessel **[57]	**Zilberman **[35]
**Right atrium**																					
Width					x		x														
Length					x																
Area					x																
**Tricuspid valve**																					
Area																	x				
Annular diameter			x							x					x						x
MV-TV distance					x																
**Right ventricle**																					
Mid RV		x					x		x		x	x		x		x			x		
RV Base					x		x														
RV Length			x		x		x							x							
RV Area	x		x		x									x							
End-diastolic volume								x						x							
End-diastolic volume																		x			
Muscle volume								x													
Muscle volume index								x													
RV geometry																				x	
Outflow tract diameter		x		x	x									x	x		x				
Outflow tract length			x																		
**Pulmonary**																					
Valve area				x																	
Annulus			x		x	x							x		x						x
Artery			x						x						x						

MV, mitral valve; TV, tricuspid valve; RV, right ventricle.

**Table 3:** Published reference data for right heart morphology

**Measurement**	**Hanseus **[46]	**Kampmann **[14]	**Kapusta **[47]	**Lester **[39]	**Shedeed **[40]	**Wessel **[57]
Right Ventricle Anterior Wall thickness		x	x	x	x	
MV-TV distance	x					
RV geometry						x

MV, mitral valve; TV, tricuspid valve;RV, right ventricle.

**Table 4:** Published reference data of right ventricular function by 2D and 3D echocardiography

**Measurement**	**Method**	**Boettler **[45]	**Clark **[42]	**Innelli **[22]	**Jin **[23]	**Koestenberger **[26]	**Moiduddin **[13]	**Norgard **[51]	**Nunez-Gil **[28]	**Seguela **[33]	**Suleymanoglu **[6]
**RV Volume and -output**											
End-diastolic volume	3D echocardiography									x	
End-systolic volume										x	
End-diastolic volume	Ellipsoid		x								
End-systolic volume			x								
End-diastolic volume	Simpson's single-plane				x			x			x
End-systolic volume					x			x			x
Stroke volume	3D echocardiography									x	
Stroke volume	Ellipsoid		x								
Stroke volume	Simpson's single-plane				x			x			
%FAC							x				
Ejection fraction	3D echocardiography									x	
Ejection fraction	Ellipsoid		x								
Ejection fraction	Simpson's single-plane	x			x			x			
RV output	Ellipsoid		x								
TAPSE				x		x			x		

RV, right ventricle; 3D, three-dimensional; %FAC, percentage fractional area change; TAPSE, tricuspid annular peak systolic excursion.

**Table 5:** Published reference data of right ventricular function by pulsed Doppler velocities and time-intervals

**Measurement**	**Ayabakan **[15]	**Cantenotti **[59]	**Cui **[16]	**Eidem **[36]	**Eidem **[18]	**Frommelt **[19]	**Hershenson **[60]	**Innelli **[22]	**Ishii **[8]	**Kapusta **[47]	**Moiduddin **[13]	**Mori **[50]	**Roberson **[53]	**Sarnari **[32]	**Shedeed **[40]	**Singh **[41]	**Stines **[54]	**Yasuoka **[34]	**Zhendong **[58]
Tricuspid systolic annular acceleration						x													
Tricuspid deceleration time						x													
RV myocardial performance index		x		x	x				x				x						
Tricuspid E velocity		x			x		x	x		x		x			x			x	x
Tricuspid A velocity		x			x		x	x		x		x			x		x	x	x
E: A ratio		x			x			x	x	x					x		x	x	x
A: E ratio												x							
E and A VTI																x	x		
A VTI																	x		
E flow velocity integral																		x	
A flow velocity integral																		x	
Tricuspid inflow area																		x	
% Atrial fraction																	x		
Presence of TR									x										
RV outflow velocity								x											x
RV VTI																x			
Peak tricuspid velocity										x									x
Late diastolic velocity and VTI	x																		
Peak systolic flow and VTI	x																		
Peak diastolic flow and VTI	x																		
Peak reverse atrial flow - R wave on ECG	x																		
R wave on ECG – peak diastolic flow	x																		
Peak diastolic flow - peak reverse atrial flow	x																		
RV isovolumic times				x					x		x		x						
RV systolic duration														x					
RV diastolic duration														x					
RV systolic duration: RV diastolic duration														x					

RV, right ventricle; E, early diastole/diastolic; A, late diastole/diastolic; VTI, velocity time integral; %, percentage; TR, tricuspid regurgitation; ECG, electrocardiograph.

**Table 6:** Published reference data of right ventricular function by tissue Doppler and strain

**Measurement**	**View**		**Sample volume**	**Boettler **[45]	**Cantenotti **[59]	**Cui **[16]	**Eidem **[36]	**Eidem **[18]	**Frommelt **[19]	**Goebel **[37]	**Harada **[21]	**Hershenson **[60]	**Hui **[44]	**Innelli **[22]	**Kapusta **[47]	**Koestenberger **[25]	**Kutty **[27]	**Matsui **[49]	**Moiduddin **[13]	**Mori **[50]	**Rafeiyian **[30]	**Roberson **[31]	**Shedeed **[40]	**Stines **[45]	**Van der Hulst **[55]	**Weidemann **[56]
RV dyssynchrony/delay											x	x												x		
RV Tissue displacement									x									x								
**Tissue Doppler velocites and time-intervals**																										
Sa; Ea; Aa; Ea: Aa; Aa: Ea	A4C	TV annulus		x			x	x	x	x	x		x	x	x				x	x	x	x	x	x		x
Sa; Ea; Aa; Ea: Aa; Ea: Sa	PLAX	RV anterior wall												x												
Sa	PSAX	RV outflow tract																						x		
Isovolumic times					x	x	x	x	x				x				x	x		x	x	x				
Deceleration time																	x			x						
Systolic and diastolic times			x		x																x					
Myocardial performance index				x	x						x							x			x	x				
E (pulsed Doppler): Ea ratio				x			x				x		x						x							
**Strain**																										
Ss, Se, Sa	A4C	RV free wall	x															x							x	
RV inflow	SC RAO	RV inferior wall															x									
RV outflow	SC RAO	RV free wall															x									
Pulmonary annulus	SC RAO	RV free wall															x									
Ss, Se, Sa	A2C	RV inferior wall																							x	
Se and Sa duration		RV free wall	x																							
% RV Strain									x									x								
RV Time to peak strain																		x						x		
**Strain rate**																										
Right atrial strain																x										
RV strain	A4C	RV free wall	x						x									x							x	
RV strain	A2C	RV inferior wall																							x	

RV, right ventricle; Sa, peak systolic velocity; Ea, early diastolic velocity; Aa, late diastolic velocity; A4C, apical four-chamber; TV, tricuspid valve; PLAX, parasternal long-axis; PSAX, parasternal short-axis; E, early diastolic velocity by pulsed Doppler; Ea, tissue Doppler early diastolic myocardial velocity; Ss, peak systolic strain; Se, peak early diastolic strain; Sa, late diastolic peak strain; SC RAO, subcostal right anterior oblique; A2C, apical two-chamber.

**Table 7:** P**ublished reference data for right heart haemodynamics**

**MMeasurement**	**Method**	**Cantenotti **[59]	**Cui **[16]	**Daubeney **[17]	**Eidem **[36]	**Eidem **[18]	**Hershenson **[60]	**Innelli **[22]	**Ishii **[8]	**Kampmann **[14]	**McQuillan **[43]	**Mori **[50]	**Pettersen **[29]	**Roberson **[53]
**Time intervals**														
PV ejection period	CW/PW Doppler				x			x	x					x
	Tissue Doppler		x											
PV pre-ejection period	CW/PW Doppler				x									
**Pulmonary artery diameter**														
PA diameter	PSAX			x									x	
PA diameter	Unspecified									x				
**Right atrial pressures**														
E/Ea		x				x	x	x				x		
**RV Systolic pressures**														
RV-RA gradient											x			
**Pulmonary arterial systolic pressures**														
PASP	RAP 10 mmHg										x			
**Inferior VENA CAVA**														
IVC % collapse								x						
IVC diameter								x						

PV, pulmonary valve; CW, continuous wave; PW, pulsed wave; PA, pulmonary artery; PSAX, parasternal short-axis; E, early diastolic velocity; Ea, tissue Doppler early diastolic myocardial velocity; RV, right ventricle; RA, right atrium; PASP, pulmonary artery systolic pressure; RAP, right atrium pressure; IVC, inferior vena cava; %, percentage.

There were insufficient published data for the following measurements: RA size (width, length and area) for children younger than ten years, tricuspid valve (TV) area, RV dimensions (base, mid and length) and -areas (diastolic and systolic), the RV fractional area change and the RV-to-RA peak pressure gradient. There were no published reference data for the RV mid-cavity dimensions presented as a sub-group for children younger than ten years; PA peak pressure gradient for children older than 12 years; inferior vena cava (IVC) diameter and percentage collapse for children younger than ten years. None of the studies estimated PA systolic pressure using an estimate of RA pressure that was based on the dimension and percentage collapse of the IVC. Lastly, there were no published data for RV volumes and ejection fraction using the area-length method.

## Discussion

While performing this review, we recognized several limitations of available reference data in paediatric echocardiography, including a lack of technical standardization of measurements, inappropriate “normal” subjects and choice of population [6,8,9,15-35], small sample sizes [12,13,21,22,33,37,49,51],[56,60] and heterogeneous methods of reporting reference values. A valid meta-analysis could not be performed because of the variability in the populations studied, in the methodology for performing and normalizing measurements, and in ways to express normalized data. These limitations were also reported in a previous review [61].

We consider this review as the most complete report on the availability of reference data in children and young adults, highlighting areas lacking sufficient data with respect to measurements of the RA, the TV, RV size and -function, the pulmonary valve (PV), the PA, and right heart haemodynamics.

### Right atrium

We identified only one publication presenting reference data for RA size for children younger than 10 years of age. In 1988, Hanseus *et al.* published a set of reference values of the RA on a sample of 120 healthy infants and children, aged three days to 15.5 years. His RA measurements included the width, length and area for the RA. He presented the data for the entire sample according to body surface area(BSA) using regression equations and nomograms [46].

Innelli *et al.* published a set of reference values for RA width on a sub-group of 40 healthy children and adolescents, aged 10-19 years. In this recent publication, reference values for the entire sub-group, aged 10-19 years were presented as one value [22].

### Tricuspid valve area

Sufficient data exist on the diameter of the TV annulus, but we discovered only one publication presenting reference data on the TV area. Singh *et al.* published reference values on the TV area based on a reference population aged two months to 50 years. The data for participants less than 16 years were presented in 3 different age-categories [41].

### Right ventricle size and/or volume

We identified one publication presenting reference data for RV basal diameter for children younger than 10 years of age [46]. This two decades old study obtained a reference population of 120 healthy infants and children, aged 3 days to 15.5 years. The data were adjusted for BSA, using regression equations and nomograms [46]. Innelli *et al.* recently published reference values on a sub-group of 40 healthy children and adolescents, aged 10-19 years as one value [22].

Two publications exist on reference values of the RV mid diameter measured in the apical four-chamber view [22,51]. Norgard *et al.* presented reference values for RV mid diameter on only 15 individuals, aged 6-16 years as one value for the entire sample [51]. More recently, Innelli *et al.* published reference values of a sub-group of 40 healthy children and adolescents, aged 10-19 years as one value [22]. We found no publications presenting reference values for RV mid diameter measured in the apical four-chamber view for children younger than 10 years.

Two publications were found containing reference values of the RV end-systolic area measured in the apical four-chamber view [45,51]. The usefulness of both of these publications however, is limited as they both present the data for the entire sample spanning more than 10 years as one value. One study had only 15 individuals, aged 6-16 years [51]. Boettler *et al.* published reference values of a larger series containing 129 reference individuals, aged 1 day to 16.9 years [45].

### Right ventricular function: % fractional area change and isovolumic acceleration

Only one publication was identified for RV % fractional area change in a sample of healthy children [13]. The sample of healthy children was a control-group consisting of 13 healthy individuals. One of the objectives of the study was to compare quantitative measurements of the RV in single RV’s to normal RV’s. The % fractional area change of the control-group consisting of 13 healthy individuals was presented. The mean age of the healthy control-group was 5.7 ± 1.8 years [13]. A study aiming to generate reference values for RV fractional area change, containing a larger sample with greater age-span and categorization may be more useful in clinical practice.

We did not find any existing reference data on isovolumic acceleration.

### Pulmonary artery peak pressure gradient

Zhendong *et al.* published reference values of the PA peak pressure gradient in 1998 in a reference population consisting of 88 healthy individuals, aged 3-12 years. The sample was divided into two age-categories [58]. No publications were found containing data for children older than 12 years.

### Right-sided haemodynamics

McQuillan *et al.* have published the only reference data on right-sided haemodynamics for children and adolescents that we could find [43]. This study of 856 participants younger than 20 years published reference data for the TV RV-RA gradient. They presented estimated PA systolic pressure values assuming that the RA pressure for all reference individuals was 10 mmHg. The reference data were presented graphically, normalised to BSA [43]. Pena *et al.* published reference data for the tricuspid regurgitation (TR) peak velocity, but of 55 new-born infants recently [52]. The authors also presented estimated PA systolic pressure values assuming that the RA pressure for all reference individuals was 5 mmHg.

No reference values for the diameter and percentage collapse of the IVC for children younger than 10 years were found. Innelli *et al.* published reference values for the IVC diameter and percent IVC collapse in a sub-group consisting of 40 healthy children and adolescents but presented the data for the entire sample, aged 10-19 years as one value [22].

No reports were found on reference values for estimated PA systolic pressure with an estimate of RA pressure on the basis of IVC size and collapse.

We found sufficient reference data for the following variables: TV annulus, RV length, RV end-diastolic area, RV outflow-tract dimension, PV diameter, PA diameter, tricuspid annulus peak systolic excursion (TAPSE) and tissue myocardial velocities at the lateral tricuspid annulus.

### Tricuspid valve annular size

Four authors have published reference data of the annulus of the TV based on reference populations ranging from infants to young adults, normalised to BSA [17,24,29,35]. The oldest publication was by King *et al.* in 1985. The most recent publication was by Pettersen *et al.* in 2008. The sample sizes ranged from the smallest by King (N = 103) to the largest by Pettersen (N = 782).

### Right ventricular size: length

Four existing publications presented reference values for the RV length, measured in the apical four-chamber view [17,22,46,51]. Hanseus *et al.* (N = 120) and Daubeney *et al.* (N = 125) presented data for the entire sample (birth to adolescent age), according to BSA whereas Inneli *et al.* (N = 40, 10-19years) and Norgard *et al.* (N = 15, 6-16years) presented data for the entire sample, as one value [17,22,46,51].

### Right ventricular size: end-diastolic area

Four authors published reference values for the RV end-diastolic area, measured in the apical four-chamber view [17,45,46,51]. Authors Hanseus *et al.* (1988, N = 120) and Daubeney *et al.* (1999, N = 125) presented data for the entire sample (birth to adolescent age), normalised to BSA [17,46]. Boettler *et al.* (2005, N = 120, 0-17years) and Norgard *et al.* (1992, N = 15, 6-16years) presented data for the entire sample, as one value [45,51].

### Right ventricular size: outflow tract dimension

There are six existing publications reporting the dimension of the RV outflow tract, measured anterior to the aortic valve in the parasternal short-axis view [9,20,29,41,46,51]. The oldest publication dates back to 1988 [46] and the most recent publication was published in 2008 [29]*.* The sample sizes range from 15 [51] to 782 [29]. The ages of the samples range from birth to adolescent ages. Four authors presented data according to BSA [9,20,29,46]. Singh *et al.* divided the sample into 3 age-categories for children younger than 16 years [41]. Norgard and Vik-Mo published reference data for the entire sample (aged 6-16years) as one value [51].

### Pulmonary valve diameter

Six publications reported reference values for the pulmonary valve PV diameter in children and adolescents [17,29,35,38,46,49]. Hanseus *et al.*, Daubeney *et al.*, Pettersen *et al.* and Zilberman *et al.* reported reference values for PV diameter in samples of healthy children ranging from birth to adolescent age and presented the data according to BSA [17,29,35,46]. Ichida *et al.* presented a set of reference intervals for children aged from birth to 15 years stratified by different age-categories [38]. Matsui *et al.* presented the measured PV diameter in a sample of 22 healthy children, aged 0-11 years as one reference interval [49]. The oldest study dates back to 1987 [38] while the most recent publication was in 2008 [29]. The sample sizes for these publications range from the 22 to 782.

### Pulmonary artery diameter

Daubeney *et al.*, Pettersen *et al.* and Kampmann *et al.* published reference data on the diameter of the PA in children ranging from infancy to adolescent age [14,17,29]. The sample sizes were 125, 782 and 2036 respectively. All three authors presented the results according to BSA.

### Right ventricular function: tricuspid annulus peak systolic excursion

Three recent publications detailed reference values for tricuspid annulus peak systolic excursion (TAPSE) in children [22,26,28]. The first publication by Innelli *et al.* presented the TAPSE values for a sub-group of 40 children and adolescents, aged 10-19 years [22]. The second publication by Koestenberger *et al.* presented the TAPSE values in a large sample (N = 640) of infants, children and adolescents [26]. The third publication by Nunez *et al*. presented the TAPSE values in a sample of 405 infants, children and adolescents [28]. The data were presented according to BSA and age-categories.

### Right ventricular function: tissue myocardial velocities at the lateral tricuspid annulus

There are 16 publications containing reference values for tissue myocardial velocities of the lateral tricuspid annulus in the apical four-chamber view [18,19,21,22,25,30,31,34],[37,40,47,50,54,55,59,60]. The sample size for these publications ranged from 16 [54,60] to 860 [25]. Seven studies presented data for the entire sample as a single value [21,22,37,40,47,54,60]. Koestenberger *et al.*[25] and Roberson *et al.*[31] presented reference data according to BSA.

## Conclusions

In the clinical diagnosis and management of patients with cardiopulmonary disorders, arrhythmogenic right ventricular dysplasia and congenital heart disease, the evaluation of the RV is of major importance.

Echocardiography is considered a cornerstone in the diagnosis and management of RV failure. Moreover, it is a leading technology, is less costly, is non-invasive and offers the advantages of wide applicability and availability when compared with other imaging modalities.

Clinically reliable reference data are lacking for important echocardiographic measurements of the RV in children and adolescents. There were insufficient published data of the following variables: RA size (width, length and area) for children younger than 10 years, TV area, RV dimensions (base, mid and length) and areas (diastolic and systolic), the RV percentage fractional area change and the RV-to-RA peak pressure gradient. There were no published reference data for the RV mid-cavity dimensions presented as a sub-group for children younger than 10 years; RV volumes and ejection fraction using the area-length method; PA peak pressure gradient for children older than 12 years; and IVC diameter and -percentage collapse for children younger than 10 years. None of the studies estimated systolic PA pressure using an estimate of RA pressure that was based on the dimension and percentage collapse of the IVC.

## Abbreviations

RV: Right ventricle / right ventricular; RA: Right atrium / right atrial; PA: Pulmonary artery / pulmonary arterial; TV: Tricuspid valve; IVC: Inferior vena cava; PV: Pulmonary valve; BSA: Body surface area; TR: Tricuspid regurgitation; TAPSE: Tricuspid annulus peak systolic excursion.

## Competing interests

The authors declared that they have no competing interests.

## Authors’ contributions

BMM conceived of the study and agreed to oversee its design and coordination, together with MEE and JCS. Under the guidance of MEE, CEL obtained and screened all the articles for this review. CEL extracted and summarized the data and drafted the manuscript. BMM, MEE and JCS revised the manuscript critically for important intellectual content and gave final approval of the version to be published. All authors read and approved the final manuscript.
